# Environmental and occupational respiratory diseases – 1046. Lung function among the workers exposed to rubber factory in West Bengal

**DOI:** 10.1186/1939-4551-6-S1-P45

**Published:** 2013-04-23

**Authors:** Naren Pandey, Kaushik Chakraborty, Ramendra Nath Mitra, Saurabh Kole, Animesh Deb, Surendra Prasad Singh, Ashim Kumar Sarkar

**Affiliations:** 1 Allergy & Asthma, Mediland Diagnostic Institute, India; 2 Population Health, Barrackpore Population Health Research Foundation, India; 3 Medicine, Life Style Clinicm, India

## Background

Exposure to dust or fumes can cause a verity of lung problems, including chronic airflow obstruction.

## Methods

The study was carried out on the 256 workers exposed to dust at the rubber factory. 16 workers with moderate exposure, 240 Workers with high exposure, spirometry (FVC, FEV1) were performed. Information on occupational history, duration of exposure, smoking habits, alcohol consumption, respiratory symptoms (breathlessness, cough and rhinitis) and self reported symptoms with disease were collected. By employing multiple linear regression modeling the potentially confounding effects of age, sex and body mass index were also incorporated into the analysis. Odds ratio were calculated for FVC<80% predicted in different exposure subgroups.

## Results

Statistically, significant reduction in FVC, FEV1 and PEFR were found when compared to age, small airway obstruction, and also in shortness of breathing. Small airway obstructions were found in dust fume (27.2%), Smoking (30.3%), Alcohol (29.3%). Lung function indices were found to be reduced with increasing duration of exposure to working environment. The FVC of the workers exposed to factory with a mean of 3.6 ± 0.6. The FEV1, for workers exposed with a mean of 2.4 ± 0.6. The mean value of the ratio of FEV1/FVC in exposed workers was 76.8 ± 8.2 there was no statistical difference between these two means.

## Conclusions

Due to high ambient dust concentration and the observed adverse effects on lung functions worker exposed to dust have more respiratory symptoms and a grater risk of airflow obstruction. A reduction of dust exposure and secondary preventive measure is advised.

